# Current status of granulocyte–macrophage colony-stimulating factor in the immunotherapy of melanoma

**DOI:** 10.1186/2051-1426-2-11

**Published:** 2014-05-13

**Authors:** Howard L Kaufman, Carl E Ruby, Tasha Hughes, Craig L Slingluff

**Affiliations:** 1 Rutgers Cancer Institute of New Jersey, 195 Little Albany Street, New Brunswick, NJ 08901, USA; 2 Rush University Medical Center, 600 S Paulina St Suite 527, Chicago, IL 60612, USA; 3 University of Virginia, P.O. Box 800709, Charlottesville, VA 22908, USA

**Keywords:** Granulocyte macrophage-colony stimulating factor, GM-CSF, Melanoma, Cellular immunotherapy

## Abstract

In 2012, it was estimated that 9180 people in the United States would die from melanoma and that more than 76,000 new cases would be diagnosed. Surgical resection is effective for early-stage melanoma, but outcomes are poor for patients with advanced disease. Expression of tumor-associated antigens by melanoma cells makes the disease a promising candidate for immunotherapy. The hematopoietic cytokine granulocyte–macrophage colony-stimulating factor (GM-CSF) has a variety of effects on the immune system including activation of T cells and maturation of dendritic cells, as well as an ability to promote humoral and cell-mediated responses. Given its immunobiology, there has been interest in strategies incorporating GM-CSF in the treatment of melanoma. Preclinical studies with GM-CSF have suggested that it has antitumor activity against melanoma and can enhance the activity of anti-melanoma vaccines. Numerous clinical studies have evaluated recombinant GM-CSF as a monotherapy, as adjuvant with or without cancer vaccines, or in combination with chemotherapy. Although there have been suggestions of clinical benefit in some studies, results have been inconsistent. More recently, novel approaches incorporating GM-CSF in the treatment of melanoma have been evaluated. These have included oncolytic immunotherapy with the GM-CSF–expressing engineered herpes simplex virus talimogene laherparepvec and administration of GM-CSF in combination with ipilimumab, both of which have improved patient outcomes in phase 3 studies. This review describes the diverse body of preclinical and clinical evidence regarding use of GM-CSF in the treatment of melanoma.

## Introduction

In 2012, it was estimated that 9180 people in the United States would die from melanoma and more than 76,000 new cases would be diagnosed [1]. The primary treatment for melanoma, excision of the malignancy, is highly effective in early-stage disease but is not a meaningful option for metastatic disease except in patients with solitary metastases or limited-volume disease [2]. For patients with advanced disease, cytotoxic chemotherapy has a limited role and curative potential in <1% of patients. For BRAF-mutant patients, treatment with vemurafenib, dabrafenib, and/or tremetinib have high rates of objective response, but median duration of response is typically ~6–8 months, after which rapid disease progression is common [2]. However, some patients with melanoma respond to immunotherapy with durable responses, and thus immunotherapy shows substantial promise for further improving durable control of advanced melanoma.

Expression of tumor-associated antigens by melanoma cells makes the disease a promising candidate for immunotherapy [3]. The potential for immunotherapy in melanoma has been demonstrated by improved outcomes among patients with stage III melanoma receiving interferon-α2b [4] and patients with metastatic melanoma receiving the anti-cytotoxic T-lymphocyte antigen 4 (CTLA-4) antibody ipilimumab alone or in combination with a gp100 peptide vaccine or dacarbazine [5,6], as well as by durable complete responses with high-dose interleukin-2 [7] and high response rates after adoptive T cell transfer therapies [8].

The hematopoietic cytokine granulocyte–macrophage colony-stimulating factor (GM-CSF) has been investigated as a monotherapy, and as a component of combination therapies for melanoma. Preclinical evidence has suggested that GM-CSF may have antitumor effects, but results from clinical trials evaluating GM-CSF present a complex picture. This review evaluates evidence regarding use of GM-CSF in melanoma and potential future strategies in this setting.

## Review

### Immunobiology of GM-CSF

**Figure 1:**
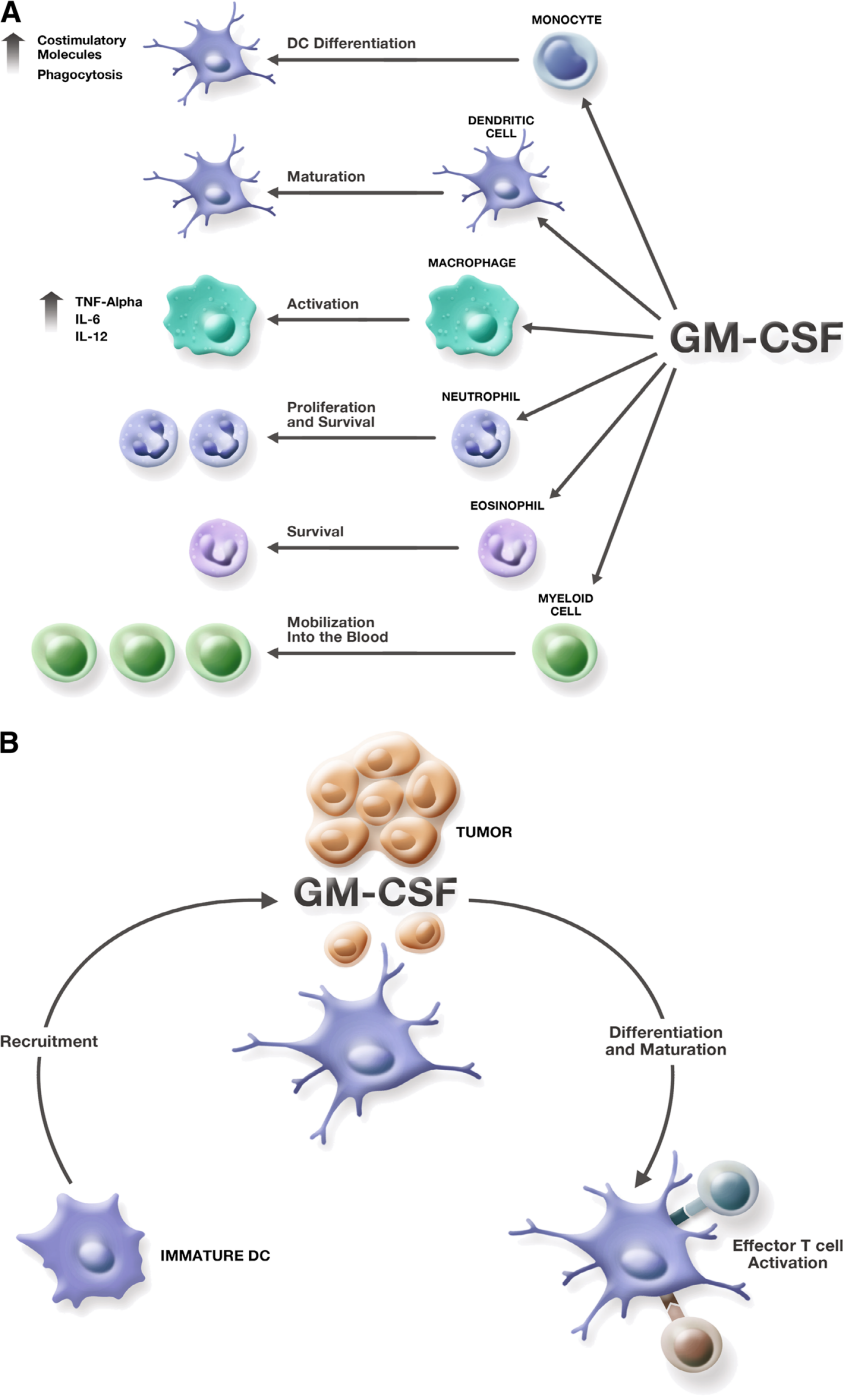
**Immunobiologic effects of GM-CSF. (A)** Effects of GM-CSF on cells of the immune system. **(B)** Effects of GM-CSF on dendritic cells and T cells in the tumor microenvironment.

GM-CSF was identified as a factor responsible for expansion and activation of granulocytes and macrophages, but has since been found to have many direct and indirect effects on multiple cell types, including cell proliferation, maturation, and survival (Figure 1A) [9]. GM-CSF plays a critical role in development and maturation of dendritic cells (DCs) and proliferation and activation of T cells, linking the innate and acquired immune response [10]. In mice, increased numbers of eosinophils, monocytes, macrophages, and lymphocytes were observed in the draining lymph node in response to treatment with irradiated melanoma cells expressing GM-CSF, resulting in a sustained antitumor response [11]. GM-CSF has also been shown to favor expansion of DC1 populations [12,13] and to increase DC-mediated responses to tumor cells (Figure 1B) [14]. In vitro studies using human myeloid leukemia cells suggest that, in addition to promoting antigen presentation, GM-CSF directs these cells toward a DC phenotype [15,16]. The role of GM-CSF in neutrophil proliferation and survival led to its use in amelioration of neutropenia following induction chemotherapy in elderly patients with acute myeloid leukemia [17].

### Studies evaluating GM-CSF in preclinical models of melanoma

The immune adjuvant properties of GM-CSF led to numerous preclinical studies assessing the ability of GM-CSF to inhibit tumor growth and/or mediate tumor regression. In a seminal study, a panel of recombinant retroviral vectors expressing various cytokines, co-stimulatory molecules, or adhesion molecules were used to infect murine B16 melanoma cells. Infected cells were then irradiated and injected subcutaneously into immune-competent hosts, followed by a subsequent challenge with wild-type B16 cells [11]. The GM-CSF–secreting tumor vaccines conveyed 90% protection, whereas vaccines expressing interleukin-2 and interferon-γ failed to mediate antitumor protection [11]. Additionally, analysis of the vaccination site revealed an influx of dividing monocytes and granulocytes, with a coincident increase in lymphocytes in tumor-draining lymph nodes, suggesting direct augmentation of antigen presentation and T-cell priming against the tumor [11].

Exogenously administered GM-CSF has been shown to augment antitumor immunity Mice immunized with an HIV envelope peptide vaccine plus GM-CSF exhibited increased cytotoxic T lymphocyte (CTL) activity compared with vaccine alone or vaccine with interleukin-2 [18]. Studies using a tumor-associated antigen vaccine (neu), in combination with GM-CSF, produced increased neu-specific antibodies alongside an enhanced CTL response [19,20]. However, tumor protection was ultimately dependent on a cell-mediated response since depletion of CD8^+^ T cells abrogated tumor regression.

Genetically modified vaccines can generate protective anti-melanoma immune responses in animal models [21]. B16 tumor lines expressing bioactive levels of murine GM-CSF have been generated and assessed [11,21,22]. Levels of GM-CSF at the site of GM-CSF–expressing tumor transplantation remain elevated for days, whereas GM-CSF dissipated rapidly after injection of irradiated tumor with recombinant GM-CSF [22]. The pharmacokinetic longevity associated with the vaccine correlated with both increased DC infiltration and tumor protection against wild-type tumors [22].

Oncolytic immunotherapy with modified herpes simplex viruses (HSV) and vaccinia viruses expressing GM-CSF have also shown promise. These vectors have elicited tumor responses in mice when injected directly into tumor lesions [23-25]. In a murine melanoma model, a temperature-sensitive strain of HSV encoding murine GM-CSF significantly reduced Harding-Passey melanoma tumor growth and improved survival of tumor-bearing mice [25]. Similarly, preclinical studies demonstrated a potent lytic effect against lesions injected with the GM-CSF–expressing oncolytic HSV talimogene laherparepvec (T-VEC; formerly Oncovex^GM-CSF^) [26]. Notably, when virus expressing GM-CSF was employed, regression of uninjected, distant lesions was enhanced compared with a regression observed with a control virus not expressing GM-CSF.

The combination of GM-CSF–secreting tumor vaccines with other immunotherapies is another potentially promising approach. Antibodies that block the co-inhibitory T-cell molecules CTLA-4 and PD-1 plus GM-CSF have boosted immune responses against melanomas [5,27-31]. Additionally, activation of co-stimulatory molecules (CD80, CD86, CD137), exogenous cytokine administration (interleukin-2, interferon-γ), and blockade of tumor angiogenesis can inhibit melanoma progression when combined with GM-CSF–expressing vaccines [32-36]. These preclinical studies strongly suggest that the combination of GM-CSF–based tumor vaccines with immunomodulatory agents has potential for clinical use.

### Clinical trials evaluating exogenously administered GM-CSF in patients with melanoma

Given the evidence of antitumor activity in preclinical models of melanoma, there has been interest in using GM-CSF to improve outcomes in the clinical setting. Numerous studies have evaluated use of recombinant GM-CSF in completely resected stage III/IV melanoma patients. Data from these studies, however, have been inconsistent.

#### GM-CSF as an adjuvant therapy

GM-CSF has been evaluated as an adjuvant, systemic monotherapy following regional lymphadenectomy, to prevent or to delay recurrence in high-risk stage III patients (Table 1). In a phase 2 study, there were statistically significant improvements in survival among patients with stage III/IV disease (*P* = 0.04 and *P* < 0.001, respectively) receiving GM-CSF (125 μg/m^2^ daily for 14 consecutive days in 28-day cycles up to 1 year) compared with historical controls [37]. A subsequent single-arm study using the same dosing regimen evaluated treatment with GM-CSF over 3 years and reported a 5-year survival rate of 60%, with 5-year disease-free survival (DFS) of 67% and 40% for patients with stage III and IV disease, respectively [38]. The effect of GM-CSF on DC has been proposed as a mechanism for supporting antitumor immunity; consistent with this hypothesis, treatment with recombinant GM-CSF has increased mature DCs in melanoma patients [39]. A randomized study of recombinant GM-CSF (125 μg/m^2^ daily for 14 consecutive days in 28-day cycles) versus placebo in patients with completely resected stage IIIB/IIIC/IV or mucosal melanoma, there was a trend toward improvement in DFS although it did not reach statistical significance (11.5 vs 9.2 months; HR, 0.88; *P* = 0.14), and no improvement in overall survival (OS; 69.6 vs 62.4 months; HR, 0.96; *P* = 0.78) [40]. However, an improvement in DFS (HR, 0.74; *P* = 0.04) and a trend toward improved OS (HR, 0.72; *P* = 0.07) was observed in stage IV patients (n = 258) [40].

**Table 1:** Clinical studies evaluating adjuvant GM-CSF in patients with surgically resected melanoma

**Citation**	**Evaluable patients**	**GM-CSF dose schedule**	**Control**	**Clinical response**
Spitler et al. [37]	48	125 μg/m^2^ for 14 d, 28-d cycles, for 1 y	Historical	OS: 37.5 mo
Markovic et al. [41]	70 (Stage IV) 149 (Stage III)	125 μg/m^2^ for 14 d, 28-d cycles, for 1 y 125 μg/m^2^ for 14 d, 28-d cycles, for 1 y	Observation	OS: 6.6 y (GM-CSF) vs 6.8 y (control) OS: 8.6 (GM-CSF) vs 5.2 y (control)
Isla et al. [42]	24	150 mg/d for 2 y	None	DFS at 1 y: 88.8%
Elias et al. [43]	45	125 μg/m^2^ for 14 d, then IL-2 9 × 10^6^ IU/m^2^ for 4 d,/28-d cycle, ± autologous vaccine	None	DFS at 15.9 mo: 60% OS at 21 mo: 64% (21 mo follow-up)
Spitler et al. [38]	98	125 μg/m^2^ for 14 d, 28-d cycles, for 3 y	None	DFS: 1.4 y 5-y survival: 60%
Lawson et al. [40]	743	250 μg/m^2^ for 14 d, 28-d cycles, for 1 y	Placebo	OS: 62.4 mo for placebo vs. 69.6 mo for GM-CSF (HR, 0.96) DFS: 9.2 mo for placebo vs. 11.5 mo for GM-CSF (HR, 0.88)

DFS = disease-free survival; GM-CSF = granulocyte-macrophage colony-stimulating factor; HR = hazard ratio; IL-2 = interleukin-2; OS = overall survival.

#### GM-CSF as intratumoral monotherapy

Several small studies have evaluated GM-CSF administered as a monotherapy by direct injection into metastatic lesions (Table 2). Clinical responses in two of these studies were modest, with only one partial response (PR) and no complete response (CR) reported [44,45]. In contrast, a study using perilesional injection of GM-CSF (400 μg/d over 5 days) to treat metastatic melanoma described reduced lesion size in 6/7 patients and a reduction in cutaneous metastases in 5/7 patients [46]. Three patients were still alive at 5-years follow-up; a fourth died tumor-free at age 93. In these studies, there was evidence of increases in DC and T-cell counts and infiltration at injected sites, and in some cases at uninjected sites, suggesting an immunologic effect of GM-CSF injection [44-46]. Other studies using novel methods (such as aerosolization and immunoembolization) to delivering GM-CSF to melanoma metastases at sites that are not readily injectable have met with mixed results (Table 2) [47-50].

**Table 2:** Studies evaluating GM-CSF as a monotherapy in patients with advanced melanoma

**Citation**	**Evaluable patients**	**GM-CSF dose schedule**	**Route of administration**	**Clinical response**	**Observations**
Si et al. [44]	13	15–50 μg/lesion at 2 sites per patient	Intralesional	1 PR, 8 SD	Responding patients had increased T-cell and Langerhans cell infiltration of the tumor
		Site 1: 5 times daily			
		Site 2: 5 times daily then once weekly for 6 mo			
Nasi et al. [45]	16	10, 20, 40, or 80 μg/injection for 10 d	Intralesional	3 SD	Significant increase in DCs and T cells at injection sites
Vaquerano et al. [51]	1	500 μg/d for 4 d, monthly	Intralesional	1 PR	Regression of melanoma cells
Hoeller et al. [46]	7	400 μg/d for 5 d, 21-d cycle	Perilesional	6 with reduced lesion size	Increased infiltration of monocytes and lymphocytes was observed in injected and systemic sites
Ridolfi et al. [52]	14	150 μg/lesion plus IL-2 3 × 10^6^ IU for 5 d, 21-day cycle	Intralesional (GM-CSF) Perilesional (IL-2)	2 PR, 2 MR, 7 SD	Some evidence of systemic immune activation
Rao et al. [47]	14	250 μg twice daily for 7 d on alternating weeks	Aerosol delivery for lung metastases	6 SD	Upregulation of cytotoxic T lymphocytes was observed in peripheral blood
Markovic et al. [48]	35	500–2000 μg (250-μg/dose increments) twice daily on days 1–7 and 15–21, over 28 d	Aerosol delivery for lung metastases	1 PR, 5 SD	A trend toward increased immune response was observed with higher doses; MTD was not reached
Sato et al. [49]	31	25–2000 μg every 4 wk	Hepatic artery immunoembolization	2 CR, 8 PR, 10 SD	Prolonged PFS correlated with higher GM-CSF doses
Eschelman et al. [50]	52	2000 μg every 4 wk	Hepatic artery immunoembolization	5 PR, 12 SD	Trend toward increased OS with GM-CSF; prolonged OS with GM-CSF in patients with bulky metastases

CR = complete response; DC = dendritic cell; GM-CSF = granulocyte-macrophage colony-stimulating factor; IL-2 = interleukin-2; MTD = maximum tolerated dose; MR = mixed response; OS = overall survival; PD = progressive disease; PFS = progression free survival; PR = partial response; SD = stable disease.

#### GM-CSF in combination with chemotherapy

A number of early-phase clinical studies have evaluated GM-CSF in conjunction with chemotherapy (Table 3). These studies were typically small with a single-arm design and used a variety of different drug regimens and dosing schedules; thus, as a whole, they are difficult to interpret. Clinical efficacy in studies varied widely, with response rates ranging from no response to >40% [53-58]. It is worth noting that several of the studies reporting high overall response rates also reported significant increases in T-cell, DC, macrophage, or natural killer–cell populations following treatment [53,55,58]. One study evaluating a chemotherapy regimen of dacarbazine, interferon-α2b, interleukin-2, and tamoxifen with three doses of GM-CSF reported a dose–response effect with increasing exposure to GM-CSF via administration over a greater number of days (*P* = 0.016) [59].

**Table 3:** Studies evaluating GM-CSF in combination with chemotherapy in patients with advanced melanoma

**Citation**	**Evaluable patients**	**GM-CSF dose schedule**	**Other agents**	**Clinical response**
Schacter et al. [53]	40	20 μg/m^2^ once daily for 7 d every 3 wk	BCNU, CDDP, DTIC, tamoxifen, IFN-α	9 CR, 11 PR, 2 SD OS:14 mo
Gajewski et al. [60]	7	5 μg/kg for 6 d	DTIC, CDDP, IL-2, IFN-α	1 CR, 1 PR, 2 MR
Gibbs et al. [61]	60	250 μg/m^2^ for 20 d, 28-d cycle	TMZ, CDDP, IL-2, IFN-α	1 CR, 11 PR Median OS: 11 mo
Vaughan et al. [59]	19	**Arm 1**: 450 μg/m^2^ on days 4, 5, 15, and 16	DTIC, CDDP, IL-2, IFN-α, TAM	2 CR, 4 PR OS: 6.2 mo Trend toward increasing response with higher GM-CSF doses
		**Arm 2**: 450 μg/m^2^ on days 4, 5, 15, 16; 225 μg/m^2^ on days 6–10 and 17–21, 28-d cycle		
		**Arm 3**: 450 μg/m^2^ on days 4–10 and 15–21, 28-d cycle		
Gong et al. [62]	30	5 μg/kg (first 25 patients) or 450 μg/m^2^ (last 8 patients) for 6 d	DTIC, CDDP, IL-2, IFN-α	3 CR, 4 PR, 6 MR, 7 SD Median OS: 15 mo
Groenewegen et al. [63]	31	2.5 μg/kg for 10 d	DTIC, IL-2, IFN-α	4 CR, 6 PR Median OS: 8 mo 1-y survival: 22%
De Gast et al. [64]	74	2.5 μg/kg for 12 d	TMZ, IL-2, IFN-α	4 CR, 19 PR, 13 SD OS: 8.3 mo 1-y survival: 41%
Smith et al. [65]	8	125 and 250 μg/m^2^/d for 7 d every 2 wk, 28-d cycle	IL-2	0 CR, 0 PR
Fruehauf et al. [54]	10	250 μg/m^2^ for 11 d	DOX, VIN	0 CR, 5 PR Median time to progression: 8 mo
Lewis et al. [66]	71	250 μg/m^2^ for 20 d, 28-d cycle	TMZ, CDDP, IFN-α, IL-2	0 CR, 10 PR Median OS: 8.6 mo
Weber et al. [67]	31	125 μg/m^2^ for 12 d, 28-d cycle	TMZ, IL-2, IFN-α	4 CR, 4 PR, 7 SD OS: 13.1 mo
Jin et al. [55]	18	175 μg/m^2^ for 4 d, 21-d cycle	DTIC, IL-2	4 CR, 8 PR
O’Day [56]	131	**Induction**: 500 μg/d for 10 d or once daily until ANC >5000/μL	**Induction**: VBL, CDDP, DTIC, IL-2, IFN-α	10 CR, 47 PR, 38 SD Median OS: 13.5 mo 1-y survival: 57%
		**Maintenance**: 250 μg/d for 14 d	**Maintenance**: IL-2	
Gunturu et al. [68]	18	250 μg/m^2^ from day 8 until AGC >5000 cells/μL on 2 consecutive days	CTX, FLU, MESNA, IL-2	1 CR, 3 PR
Locke et al. [57]	14	250 μg/m^2^ until WBC >30000/μL or for 10 d, 21-d cycle	OX, DOX	0 CR, 0 PR, 5 SD
Lutzky et al. [69]	30	125 μg/m^2^ for 35 d	IL-2	0 CR, 4 PR, 8 SD Median OS: 10.7 mo 1-y survival: 32.5%

AGC = absolute granulocyte count; ANC = absolute neutrophil count; BCNU = carmustine; CDDP = cisplatin; CR = complete response; CTX = cyclophosphamide; DOX = docetaxel; DTIC = dacarbazine; FLU = fludarabine; GM-CSF = granulocyte-macrophage colony-stimulating factor; IFN-α = interferon-α; IL-2 = interleukin-2; MESNA = sodium 2-mercaptoethanesulfonate; MR = mixed response; OS = overall survival; OX = oxaliplatin; PFS = progression-free survival; PR = partial response; SD = stable disease; TAM = tamoxifen; TMZ = temozolomide; VBL = vinblastine; VIN = vinorelbine.

#### GM-CSF as an adjuvant with cancer vaccines

Given the significant body of evidence from preclinical studies [11,18,20,21,70], GM-CSF has been evaluated as an adjuvant to cancer vaccines in a number of clinical studies. Several approaches to administering GM-CSF as an adjuvant have been employed, including coadministration with the vaccine [71-74], injection at the vaccination site [75,76] systemic administration [77,78], and administration of a plasmid/viral vector encoding GM-CSF [79,80]. The dose of GM-CSF administered as an adjuvant is typically less than the recommended overall weekly dose of 250 μg/m^2^/day for 21 days for use in myeloid reconstitution after autologous bone marrow transplantation [17].

In contrast to data from murine studies, the adjuvant effect of GM-CSF in human trials is inconsistent. In a study that evaluated coadministration of GM-CSF with multipeptide (including gp100 and tyrosinase peptides) melanoma vaccines incorporating GM-CSF and incomplete Freund’s adjuvant (IFA) in patients with advanced melanoma, there was a high T-cell response rate and a correlation between T-cell reactivity to the melanoma peptides and clinical outcome [72]. GM-CSF combined with IFA as an adjuvant for a vaccine containing 12 melanoma peptides resulted in a similar immunologic response in patients with resected stage III/IIIB/IV melanoma [74]. Similarly, systemic administration of GM-CSF following a peptide vaccination augmented T-cell response in three patients with advanced melanoma [77]. However, in a study that evaluated intradermal vaccination with tyrosinase peptides followed by intradermal GM-CSF, detectable T-cell responses were observed in only 4/15 (27%) evaluable patients [76].

Although results from these studies have suggested that administration of adjuvant GM-CSF might improve immune responses, none included control groups. Recently, clinical trials including controls have evaluated effects of GM-CSF administered locally at the vaccination site (Table 4). In a phase 1 study comparing different adjuvant strategies, vaccination with tyrosinase peptide plus GM-CSF or keyhole limpet hemocyanin (KLH) did not induce greater immune responses compared with vaccination with peptide alone, although combination with GM-CSF plus KLH had a moderate adjuvant effect [75]. Two recent randomized prospective trials suggested that addition of GM-CSF to melanoma vaccines did not improve cellular immune responses and, indeed, may have had negative effects [71,81]. Notably, both studies combined GM-CSF with another adjuvant (IFA or BCG) which may have influenced the immune response. The inconsistent effect of GM-CSF on immune responses to vaccines may be due in part to competing effects inducing dendritic cell maturation on one hand, and inducing myeloid suppressor cells on the other. A recent phase 2, randomized controlled trial (E1696) evaluated treatment of advanced melanoma with multipeptide vaccine alone or with subcutaneous interferon-α, GM-CSF, or interferon-α plus GM-CSF [78,82]. Consistent with results of studies evaluating administration of GM-CSF at the vaccine site, no significant improvement in T-cell or clinical response was observed with interferon-α and/or GM-CSF administration with vaccination [78,82].

**Table 4:** Cancer vaccines testing the adjuvant effect of GM-CSF administered locally at the site of vaccination

**Citation**	**Design (Enrollment)**	**Ag (Route)**	**GM-CSF form (Route)**	**Coadmin-istration?**	**Study design**	**Effect of GM-CSF**	**Summary effect of GM-CSF**
Scheibenbogen et al. [75]	Sequential cohorts (n = 43)	Tyrosinase peptides (ID/SC)	Protein (ID/SC) 75 μg/d x 4 d/vaccine	Yes	Sequential:	Minimal adjuvant effect Sequential trial cohorts	Minimal adjuvant effect
					1. Peptides alone		
					2. Peptides + GM-CSF		
					3. Peptides + KLH		
					4. Peptides + GM-CSF + KLH		
Slingluff et al. [81]	Randomized (n = 121)	Melanoma peptides (ID/SC)	Protein 110 μg (ID/SC)	Yes	Randomized:	Negative on CD4 and CD8 T cells; too few events to differences in survival between groups	Diminished, compared with IFA
					1. Peptides + IFA		
					2. Peptides + IFA + GM-CSF		
Faries et al. [71]	Randomized (n = 97)	Whole melanoma cell vaccine (ID)	Protein 200 μg/m^2^/d x 5 days (ID)	Yes	Randomized:	Better Ab, worse DTH; more Eos, Dec monocytes; more deaths	Diminished compared with BCG
					1. Whole cell vaccine + BCG + GM-CSF		
					2. Whole cell vaccine + BCG		
Kirkwood et al. [78]	2 × 2 (n = 120)	MART-1, gp100, and tyrosinase peptides (SC)	250 μg/d x 14 out of 28 days	Yes	2 × 2:	No effect across treatment arms on best overall response	Minimal adjuvant effect
					Arm A: Peptide Vaccine Alone		
					Arm B: GM-CSF (250 μg/d x 14 out of 28 d) + vaccine		
					Arm C: IFN-α + vaccine		
					Arm D: GM-CSF + IFN-α + vaccine		

ID = intradermal; SC = subcutaneous; IFA = incomplete Freund’s adjuvant; BCG = Bacille Calmette-Guerin.

Results from clinical studies evaluating GM-CSF as an adjuvant to melanoma vaccines suggest the biologic effects of GM-CSF are complex and can be influenced by numerous factors. GM-CSF administered with a heat shock protein vaccine has been implicated in the induction of myeloid-derived suppressor cells (MDSC) in melanoma patients [83]. On the other hand, daily subcutaneous administration of GM-CSF (125 μg/m^2^ for 14 days in 28-day cycles) increased circulating mature DC but did not increase MDSC in melanoma patients [39]. It has been suggested that negative immunologic effects of GM-CSF may be associated primarily with high doses of GM-CSF (doses of 225 μg/d or higher in melanoma patients) [84]. In a trial of a multipeptide melanoma vaccine, immune responses were lower with GMCSF plus IFA than with IFA alone (the dose used in that trial was arguably less than 20 μg/day) [81]. Thus, even low doses of GM-CSF administered with a multipeptide vaccine may have negative immunologic effects. Additional studies are also needed to determine if GM-CSF alters the function of vaccine-induced T cells and whether inclusion of GM-CSF with the vaccine may affect clinical outcome.

### Novel strategies incorporating GM-CSF

#### GM-CSF–expressing oncolytic immunotherapy

Preclinical studies have indicated an important role for GM-CSF in the tumor microenvironment and have suggested that increased expression of GM-CSF can inhibit tumor growth. However, administration of exogenous recombinant GM-CSF appears insufficient to mediate clinically meaningful improvements in outcomes in most instances. Consequently, there has been significant interest in novel treatment approaches incorporating GM-CSF.

Among the most extensively evaluated agents is the attenuated, oncolytic, herpes simplex virus, type 1 (HSV-1)–encoding human GM-CSF talimogene laherparepvec (T-VEC; formerly Oncovex^GM-CSF^) [85-88]. The vector was generated from the HSV JS1 strain and was attenuated by functional deletion of the *ICP34.5* and *ICP47* viral genes, which renders the virus nonpathogenic in normal eukaryotic cells, promotes selective replication in tumor cells, and enhances immunogenicity [26] T-VEC has also been engineered to encode human GM-CSF, which further enhances the antitumor immune response. T-VEC is proposed to have a dual mechanism of action resulting in local tumor destruction by introduction of an oncolytic virus into tumor cells and induction of a systemic antitumor immune response.

In a phase 1 study, patients with subcutaneous or cutaneous metastases from breast, gastrointestinal, head and neck, or melanoma malignancies were treated with T-VEC. Clinically stable disease was noted in 3/26 patients administered intralesional T-VEC, with no patients having CR or PR during the study period [86]. However, follow-up biopsies in 14/19 patients showed necrosis, infiltration of T cells, and the presence of replicating virus [86]. Notably, there was evidence of expression of GM-CSF in injected lesions. In a subsequent phase 2 study among 50 patients with unresectable stage IIIC/IV melanoma administered intralesional T-VEC, the overall response rate was 26% (8 CR, 5 PR) [88]. Two additional patients were rendered disease-free by surgical resection following treatment. One-year survival was 58% for all patients and 40% among patients with stage IVM1c disease. Immunologic analysis of a subset of patients enrolled in the phase 2 study confirmed the presence of both local and distant antitumor immune responses following T-VEC administration. Biopsies performed on 11 patients after their sixth intratumoral injection were compared to nonstudy patients with metastatic melanoma [87]. In general, postvaccination tumors demonstrated extensive lymphocyte infiltration. Evaluation of both injected and noninjected lesions in T-VEC–treated patients revealed a significant increase in MART-1–specific T-cell response in both tumor and distant disease sites compared with unvaccinated controls. Regulatory CD4+FoxP3+ and suppressor CD8+FoxP3+ T cells and myeloid-derived suppressor cells were evaluated in injected tumor biopsies and found to be decreased when compared to unvaccinated control patients. When primary injected lesions were compared to distant noninjected lesions, the same general phenotypic pattern of effector T-cell and Treg infiltrates was seen; a greater number of CD4+FoxP3+ and CD8+FoxP3+ cells were present at the nontarget site than at the target sites. A randomized, phase 3 study, OPTiM, comparing intralesional T-VEC to subcutaneous GM-CSF demonstrated an improvement in durable response rate (16% vs 2%, respectively) and a trend toward improved OS at interim analysis (HR, 0.79 [95% CI, 0.61–1.02]) favoring the T-VEC arm [89]. The most frequently occurring adverse events were fatigue, chills, and pyrexia [89].

#### Systemic combination immunotherapy

GM-CSF has also been combined with ipilimumab, an anti-CTLA-4 monoclonal antibody. In a multicenter, phase 2 study, patients with metastatic melanoma were randomized to treatment with ipilimumab plus GM-CSF (250 μg/d subcutaneously for 14 days in 21-day cycles) or ipilimumab alone [90]. Patients receiving combination therapy experienced a significant improvement in 1-year OS. Although there were no significant differences in overall toxicity between the treatment arms, patients receiving combination therapy had a lower rate of serious adverse events.

Several other approaches utilizing GM-CSF are being developed. These include an oncolytic vaccinia virus encoding GM-CSF [91], autologous dendritic cells and allogeneic whole tumor cells encoding GM-CSF [92-94], adjuvant GM-CSF following vaccination with peptide or RNA encoding melanoma peptides [95,96] and autologous dendritic cell or whole tumor cell vaccines [97], and GM-CSF DNA vaccines [98].

## Conclusions

GM-CSF has been studied extensively in murine models and in human clinical trials, alone and as adjuvant therapy for melanoma. There is evidence from numerous studies that GM-CSF can induce antitumor immunity when administered by a variety of different routes. Although there was initial enthusiasm for recombinant GM-CSF based on uncontrolled clinical trials in stage III/IV melanoma, therapeutic benefit has not been confirmed in larger, prospective, randomized trials. The adjuvant or combination use of GM-CSF has been more promising although results have been inconsistent and may depend on the potency of the immunotherapy regimen, GM-CSF dose, route and schedule of administration, and stage of disease. There is emerging evidence that GM-CSF may be a regulatory cytokine with the ability to promote both effector and regulatory/suppressor T cell populations. Thus, strategies that block suppressor T cell and myeloid-derived suppressor cell elements may enhance the antitumor activity of GM-CSF. GM-CSF has been particularly effective in studies of oncolytic HSV therapy of melanoma and in combination with ipilimumab. Further research into the basic biology of GM-CSF on effector and suppressor immune cells and expanded clinical studies of combination treatments will help define the full therapeutic potential of GM-CSF in treatment of melanoma.

## Abbreviations

CTLA-4: Cytotoxic T-lymphocyte antigen 4; CR: Complete response; DC: Dendritic cells; DFS: Disease-free survival; GM-CSF: Granulocyte–macrophage colony-stimulating factor; HR: Hazard ratio; HSV: Herpes simplex viruses; IFA: Incomplete Freund’s adjuvant; KLH: Keyhole limpet hemocyanin; MDSC: Myeloid-derived suppressor cells; OS: Overall survival; PD-1: Programmed death 1; PR: Partial response; T-VEC: Talimogene laherparepvec.

## Competing interests

HLK serves as a paid consultant and advisory board member for Amgen Inc. CLS is a scientific advisory board member for Immatics Biotechnologies and Polynoma LLC and a funded investigator for GlaxoSmithKline in the field of cancer vaccines; all funds from these activities are paid to the University of Virginia, not to Dr. Slingluff personally. Dr. Slingluff is an inventor on several patents for peptides used in cancer vaccines; the patents are held by the University of Virginia Licensing and Ventures Group. CER and TH have no conflicts to declare.

## Authors’ contributions

All authors participated in critical analysis of the literature and drafting of the manuscript. All authors read and approved the final manuscript.

## References

[B1] Siegel R Naishadham D Jemal A Cancer statistics, 2012 CA Cancer J Clin 2012 62 10 29 10.3322/caac.20138 22237781

[B2] National Comprehensive Cancer Network NCCN Guidelines® Version 2.2013 Melanoma 2012 Fort Washington, PA

[B3] Pandolfi F Cianci R Pagliari D Casciano F Bagala C Astone A Landolfi R Barone C The immune response to tumors as a tool toward immunotherapy Clin Dev Immunol 2011 2011 894704 22190975 10.1155/2011/894704 PMC3235449

[B4] Eggermont AM Suciu S Santinami M Testori A Kruit WH Marsden J Punt CJ Sales F Gore M Mackie R Kusic Z Dummer R Hauschild A Musat E Spatz A Keilholz U Group EM Adjuvant therapy with pegylated interferon alfa-2b versus observation alone in resected stage III melanoma: final results of EORTC 18991, a randomised phase III trial Lancet 2008 372 117 126 10.1016/S0140-6736(08)61033-8 18620949

[B5] Hodi FS O’Day SJ McDermott DF Weber RW Sosman JA Haanen JB Gonzalez R Robert C Schadendorf D Hassel JC Akerley W van den Eertwegh AJ Lutzky J Lorigan P Vaubel JM Linette GP Hogg D Ottensmeier CH Lebbe C Peschel C Quirt I Clark JI Wolchok JD Weber JS Tian J Yellin MJ Nichol GM Hoos A Urba WJ Improved survival with ipilimumab in patients with metastatic melanoma N Engl J Med 2010 363 711 723 10.1056/NEJMoa1003466 20525992 PMC3549297

[B6] Robert C Thomas L Bondarenko I O’Day S DJ M Garbe C Lebbe C Baurain JF Testori A Grob JJ Davidson N Richards J Maio M Hauschild A Miller WH Jr Gascon P Lotem M Harmankaya K Ibrahim R Francis S Chen TT Humphrey R Hoos A Wolchok JD Ipilimumab plus dacarbazine for previously untreated metastatic melanoma N Engl J Med 2011 364 2517 2526 10.1056/NEJMoa1104621 21639810

[B7] Atkins MB Lotze MT Dutcher JP Fisher RI Weiss G Margolin K Abrams J Sznol M Parkinson D Hawkins M Paradise C Kunkel L Rosenberg SA High-dose recombinant interleukin 2 therapy for patients with metastatic melanoma: analysis of 270 patients treated between 1985 and 1993 J Clin Oncol 1999 17 2105 2116 10561265 10.1200/JCO.1999.17.7.2105

[B8] Rosenberg SA Yang JC Sherry RM Kammula US Hughes MS Phan GQ Citrin DE Restifo NP Robbins PF Wunderlich JR Morton KE Laurencot CM Steinberg SM White DE Dudley ME Durable complete responses in heavily pretreated patients with metastatic melanoma using T-cell transfer immunotherapy Clin Cancer Res 2011 17 4550 4557 10.1158/1078-0432.CCR-11-0116 21498393 PMC3131487

[B9] Metcalf D Hematopoietic cytokines Blood 2008 111 485 491 10.1182/blood-2007-03-079681 18182579 PMC2200848

[B10] Hercus TR Thomas D Guthridge MA Ekert PG King-Scott J Parker MW Lopez AF The granulocyte-macrophage colony-stimulating factor receptor: linking its structure to cell signaling and its role in disease Blood 2009 114 1289 1298 10.1182/blood-2008-12-164004 19436055 PMC2727416

[B11] Dranoff G Jaffee E Lazenby A Golumbek P Levitsky H Brose K Jackson V Hamada H Pardoll D Mulligan RC Vaccination with irradiated tumor cells engineered to secrete murine granulocyte-macrophage colony-stimulating factor stimulates potent, specific, and long-lasting anti-tumor immunity Proc Natl Acad Sci USA 1993 90 3539 3543 10.1073/pnas.90.8.3539 8097319 PMC46336

[B12] Lonial S Akhtari M Kaufman J Torre C Lechowicz MJ Flowers C Sinha R Khoury HJ Langston AA Waller EK Mobilization of hematopoietic progenitors from normal donors using the combination of granulocyte-macrophage colony-stimulating factor and granulocyte colony-stimulating factor results in fewer plasmacytoid dendritic cells in the graft and enhanced donor T cell engraftment with Th1 polarization: results from a randomized clinical trial Biol Blood Marrow Transplant 2013 19 460 467 10.1016/j.bbmt.2012.11.017 23201472

[B13] Lonial S Hicks M Rosenthal H Langston A Redei I Torre C Duenzl M Feinstein B Cherry J Waller EK A randomized trial comparing the combination of granulocyte-macrophage colony-stimulating factor plus granulocyte colony-stimulating factor versus granulocyte colony-stimulating factor for mobilization of dendritic cell subsets in hematopoietic progenitor cell products Biol Blood Marrow Transplant 2004 10 848 857 10.1016/j.bbmt.2004.07.008 15570253

[B14] Mach N Gillessen S Wilson SB Sheehan C Mihm M Dranoff G Differences in dendritic cells stimulated in vivo by tumors engineered to secrete granulocyte-macrophage colony-stimulating factor or Flt3-ligand Cancer Res 2000 60 3239 3246 10866317

[B15] Choudhury BA Liang JC Thomas EK Flores-Romo L Xie QS Agusala K Sutaria S Sinha I Champlin RE Claxton DF Dendritic cells derived in vitro from acute myelogenous leukemia cells stimulate autologous, antileukemic T-cell responses Blood 1999 93 780 786 9920826

[B16] Boyer MW Waller EK Bray RA Unangst T Johnson TS Phillips C Jurickova I Winton EF Yeager AM Cytokine upregulation of the antigen presenting function of acute myeloid leukemia cells Leukemia 2000 14 412 418 10.1038/sj.leu.2401685 10720135

[B17] Leukine® (sargramostim) Full Prescribing Information 2009 Cambridge, MA: Genzyme Corporation

[B18] Ahlers JD Dunlop N Alling DW Nara PL Berzofsky JA Cytokine-in-adjuvant steering of the immune response phenotype to HIV-1 vaccine constructs: granulocyte-macrophage colony-stimulating factor and TNF-alpha synergize with IL-12 to enhance induction of cytotoxic T lymphocytes J Immunol 1997 158 3947 3958 9103465

[B19] Reilly RT Gottlieb MB Ercolini AM Machiels JP Kane CE Okoye FI Muller WJ Dixon KH Jaffee EM HER-2/neu is a tumor rejection target in tolerized HER-2/neu transgenic mice Cancer Res 2000 60 3569 3576 10910070

[B20] Reilly RT Machiels JP Emens LA Ercolini AM Okoye FI Lei RY Weintraub D Jaffee EM The collaboration of both humoral and cellular HER-2/neu-targeted immune responses is required for the complete eradication of HER-2/neu-expressing tumors Cancer Res 2001 61 880 883 11221874

[B21] Borrello I Pardoll D GM-CSF-based cellular vaccines: a review of the clinical experience Cytokine Growth Factor Rev 2002 13 185 193 10.1016/S1359-6101(01)00034-X 11900993

[B22] Simmons AD Li B Gonzalez-Edick M Lin C Moskalenko M Du T Creson J VanRoey MJ Jooss K GM-CSF-secreting cancer immunotherapies: preclinical analysis of the mechanism of action Cancer Immunol Immunother 2007 56 1653 1665 10.1007/s00262-007-0315-2 17410360 PMC11029840

[B23] Ju DW Cao X Acres B Active specific immunotherapy of pulmonary metastasis with vaccinia melanoma oncolysate prepared from granulocyte/macrophage-colony-stimulating-factor-gene-encoded vaccinia virus J Cancer Res Clin Oncol 1996 122 716 722 8954168 10.1007/BF01209118 PMC12200445

[B24] Ju DW Cao X Acres B Intratumoral injection of GM-CSF gene encoded recombinant vaccinia virus elicits potent antitumor response in a mixture melanoma model Cancer Gene Ther 1997 4 139 144 9080123

[B25] Toda M Martuza RL Rabkin SD Tumor growth inhibition by intratumoral inoculation of defective herpes simplex virus vectors expressing granulocyte-macrophage colony-stimulating factor Mol Ther 2000 2 324 329 10.1006/mthe.2000.0130 11020347

[B26] Liu BL Robinson M Han ZQ Branston RH English C Reay P McGrath Y Thomas SK Thornton M Bullock P Love CA Coffin RS ICP34.5 deleted herpes simplex virus with enhanced oncolytic, immune stimulating, and anti-tumour properties Gene Ther 2003 10 292 303 10.1038/sj.gt.3301885 12595888

[B27] Li B VanRoey M Wang C Chen TH Korman A Jooss K Anti-programmed death-1 synergizes with granulocyte macrophage colony-stimulating factor–secreting tumor cell immunotherapy providing therapeutic benefit to mice with established tumors Clin Cancer Res 2009 15 1623 1634 10.1158/1078-0432.CCR-08-1825 19208793

[B28] Quezada SA Peggs KS Curran MA Allison JP CTLA4 blockade and GM-CSF combination immunotherapy alters the intratumor balance of effector and regulatory T cells J Clin Invest 2006 116 1935 1945 10.1172/JCI27745 16778987 PMC1479425

[B29] Topalian SL Hodi FS Brahmer JR Gettinger SN Smith DC McDermott DF Powderly JD Carvajal RD Sosman JA Atkins MB Leming PD Spigel DR Antonia SJ Horn L Drake CG Pardoll DM Chen L Sharfman WH Anders RA Taube JM McMiller TL Xu H Korman AJ Jure-Kunkel M Agrawal S McDonald D Kollia GD Gupta A Wigginton JM Sznol M Safety, activity, and immune correlates of anti-PD-1 antibody in cancer N Engl J Med 2012 366 2443 2454 10.1056/NEJMoa1200690 22658127 PMC3544539

[B30] van Elsas A Hurwitz AA Allison JP Combination immunotherapy of B16 melanoma using anti-cytotoxic T lymphocyte-associated antigen 4 (CTLA-4) and granulocyte/macrophage colony-stimulating factor (GM-CSF)-producing vaccines induces rejection of subcutaneous and metastatic tumors accompanied by autoimmune depigmentation J Exp Med 1999 190 355 366 10.1084/jem.190.3.355 10430624 PMC2195583

[B31] Weber J Immune checkpoint proteins: a new therapeutic paradigm for cancer–preclinical background: CTLA-4 and PD-1 blockade Semin Oncol 2010 37 430 439 10.1053/j.seminoncol.2010.09.005 21074057

[B32] Choi KJ Kim JH Lee YS Kim J Suh BS Kim H Cho S Sohn JH Kim GE Yun CO Concurrent delivery of GM-CSF and B7-1 using an oncolytic adenovirus elicits potent antitumor effect Gene Ther 2006 13 1010 1020 10.1038/sj.gt.3302759 16525479

[B33] Li B Lin J Vanroey M Jure-Kunkel M Jooss K Established B16 tumors are rejected following treatment with GM-CSF-secreting tumor cell immunotherapy in combination with anti-4-1BB mAb Clin Immunol 2007 125 76 87 10.1016/j.clim.2007.07.005 17706463

[B34] Prell RA Li B Lin JM VanRoey M Jooss K Administration of IFN-alpha enhances the efficacy of a granulocyte macrophage colony stimulating factor-secreting tumor cell vaccine Cancer Res 2005 65 2449 2456 10.1158/0008-5472.CAN-04-1975 15781661

[B35] Stagg J Wu JH Bouganim N Galipeau J Granulocyte-macrophage colony-stimulating factor and interleukin-2 fusion cDNA for cancer gene immunotherapy Cancer Res 2004 64 8795 8799 10.1158/0008-5472.CAN-04-1776 15604233

[B36] Li B Lalani AS Harding TC Luan B Koprivnikar K Huan Tu G Prell R VanRoey MJ Simmons AD Jooss K Vascular endothelial growth factor blockade reduces intratumoral regulatory T cells and enhances the efficacy of a GM-CSF-secreting cancer immunotherapy Clin Cancer Res 2006 12 6808 6816 10.1158/1078-0432.CCR-06-1558 17121902

[B37] Spitler LE Grossbard ML Ernstoff MS Silver G Jacobs M Hayes FA Soong SJ Adjuvant therapy of stage III and IV malignant melanoma using granulocyte-macrophage colony-stimulating factor J Clin Oncol 2000 18 1614 1621 10764421 10.1200/JCO.2000.18.8.1614

[B38] Spitler LE Weber RW Allen RE Meyer J Cruickshank S Garbe E Lin HY Soong SJ Recombinant human granulocyte-macrophage colony-stimulating factor (GM-CSF, sargramostim) administered for 3 years as adjuvant therapy of stages II(T4), III, and IV melanoma J Immunother 2009 32 632 637 10.1097/CJI.0b013e3181a7d60d 19483646

[B39] Daud AI Mirza N Lenox B Andrews S Urbas P Gao GX Lee JH Sondak VK Riker AI Deconti RC Gabrilovich D Phenotypic and functional analysis of dendritic cells and clinical outcome in patients with high-risk melanoma treated with adjuvant granulocyte macrophage colony-stimulating factor J Clin Oncol 2008 26 3235 3241 10.1200/JCO.2007.13.9048 18591558

[B40] Lawson DH Lee SJ Tarhini AA Margolin KA Ernstoff MS Kirkwood JM E4697: Phase III cooperative group study of yeast-derived granulocyte macrophage colonystimulating factor (GM-CSF) versus placebo as adjuvant treatment of patients with completely resected stage III-IV melanoma J Clin Oncol 2010 28 abstr 8504

[B41] Markovic S Burch PA LaPlant B Heun JM Bradshaw R Adjuvant GM-CSF therapy for patients with resected stage III/IV melanoma: A retrospective review of a single-center experience J Clin Oncol 2011 29 abstr 8596

[B42] Isla D Filipovich E Mayordomo JI Andres R Puig S Escudero MP Gallego O Trujillo R Revenga F Alvarez I Saenz A Polo E Tres A Daily GM-CSF for patients with very high-risk resected melanoma: a pilot trial Proc Am Soc Clin Oncol 2002 21 abstr 2784

[B43] Elias EG Zapas JL McCarron EC Beam SL Hasskamp JH Culpepper WJ Sequential administration of GM-CSF (Sargramostim) and IL-2 +/−autologous vaccine as adjuvant therapy in cutaneous melanoma: an interim report of a phase II clinical trial Cancer Biother Radiopharm 2008 23 285 291 10.1089/cbr.2007.0438 18593361

[B44] Si Z Hersey P Coates AS Clinical responses and lymphoid infiltrates in metastatic melanoma following treatment with intralesional GM-CSF Melanoma Res 1996 6 247 255 10.1097/00008390-199606000-00008 8819128

[B45] Nasi ML Lieberman P Busam KJ Prieto V Panageas KS Lewis JJ Houghton AN Chapman PB Intradermal injection of granulocyte-macrophage colony-stimulating factor (GM-CSF) in patients with metastatic melanoma recruits dendritic cells Cytokines Cell Mol Ther 1999 5 139 144 10641571

[B46] Hoeller C Jansen B Heere-Ress E Pustelnik T Mossbacher U Schlagbauer-Wadl H Wolff K Pehamberger H Perilesional injection of r-GM-CSF in patients with cutaneous melanoma metastases J Invest Dermatol 2001 117 371 374 10.1046/j.0022-202x.2001.01427.x 11511318

[B47] Rao RD Anderson PM Arndt CA Wettstein PJ Markovic SN Aerosolized granulocyte macrophage colony-stimulating factor (GM-CSF) therapy in metastatic cancer Am J Clin Oncol 2003 26 493 498 10.1097/01.coc.0000037664.04141.D0 14528078

[B48] Markovic SN Suman VJ Nevala WK Geeraerts L Creagan ET Erickson LA Rowland KM Jr Morton RF Horvath WL Pittelkow MR A dose-escalation study of aerosolized sargramostim in the treatment of metastatic melanoma: an NCCTG Study Am J Clin Oncol 2008 31 573 579 10.1097/COC.0b013e318173a536 19060590 PMC2694721

[B49] Sato T Eschelman DJ Gonsalves CF Terai M Chervoneva I McCue PA Shields JA Shields CL Yamamoto A Berd D Mastrangelo MJ Sullivan KL Immunoembolization of malignant liver tumors, including uveal melanoma, using granulocyte-macrophage colony-stimulating factor J Clin Oncol 2008 26 5436 5442 10.1200/JCO.2008.16.0705 18838710 PMC6815970

[B50] Eschelman DJ Gonsalves CF Terai M Laudadio M Sullivan KL Mastrangelo MJ Sato T The results of a randomized phase II study using embolization with or without granulocyte-macrophage colony-stimulating factor (GM-CSF) in uveal melanoma patients with hepatic metastasis J Clin Oncol 2011 29 abstr 8577

[B51] Vaquerano JE Cadbury P Treseler P Sagebiel R Leong SP Regression of in-transit melanoma of the scalp with intralesional recombinant human granulocyte-macrophage colony-stimulating factor Arch Dermatol 1999 135 1276 1277 10.1001/archderm.135.10.1276 10522685

[B52] Ridolfi L Ridolfi R Preliminary experiences of intralesional immunotherapy in cutaneous metastatic melanoma Hepatogastroenterology 2002 49 335 339 11995445

[B53] Schachter J Rakowsky E Sulkes A Adler A A sequential four-drug chemotherapy and biotherapy with interferon alpha and GM-CSF–an innovative protocol for the treatment of metastatic melanoma Cancer Biother Radiopharm 1998 13 155 164 10.1089/cbr.1998.13.155 10850351

[B54] Fruehauf JP Kong KM Jakowatz JG Docetaxel and vinorelbine plus GM-CSF in malignant melanoma Oncology (Williston Park) 2005 19 19 22 15934496

[B55] Jin S Zhang Q Kang X Wang J Sun W Malignant melanoma therapy by chemotherapy and autoimmunity induced by cytokine Cancer Biother Radiopharm 2009 24 237 241 10.1089/cbr.2008.0552 19409046

[B56] O’Day SJ Atkins MB Boasberg P Wang HJ Thompson JA Anderson CM Gonzalez R Lutzky J Amatruda T Hersh EM Weber JS Phase II multicenter trial of maintenance biotherapy after induction concurrent Biochemotherapy for patients with metastatic melanoma J Clin Oncol 2009 27 6207 6212 10.1200/JCO.2008.20.3075 19917850

[B57] Locke F Clark JI Gajewski TF A phase II study of oxaliplatin, docetaxel, and GM-CSF in patients with previously treated advanced melanoma Cancer Chemother Pharmacol 2010 65 509 514 10.1007/s00280-009-1057-y 19597729

[B58] Lutzky J Weber R Nunez Y Gillett M Spitler L A phase 1 study of granulocyte macrophage colony-stimulating factor (sargramostim) and escalating doses of thalidomide in patients with high-risk malignant melanoma J Immunother 2009 32 79 85 10.1097/CJI.0b013e31818c8aaf 19307996

[B59] Vaughan MM Moore J Riches PG Johnston SR A’Hern RP Hill ME Eisen T Ayliffe MJ Thomas JM Gore ME GM-CSF with biochemotherapy (cisplatin, DTIC, tamoxifen, IL-2 and interferon-alpha): a phase I trial in melanoma Ann Oncol 2000 11 1183 1189 10.1023/A:1008348005349 11061616

[B60] Gajewski T Flickinger S A phase II study of outpatient chemoimmunotherapy using cisplatin and DTIC followed by GM-CSF, IL-2, and IFN-2b in patients (pts) with metastatic melanoma Proc Am Soc Clin Oncol 2000 19 abstr 2271

[B61] Gibbs P O’Day S Richards J Weber J Anderson C Gonzalez R Biochemotherapy (BCT) for stage IV melanoma incorporating temozolomide, decrescendo interleukin-2 (IL-2) and GM-CSF Proc Am Soc Clin Oncol 2000 19 2255 10.1081/cnv-58832 16100942

[B62] Gong I-Y Swiger S Gajewski T Integration of GM-CSF into outpatient chemoimmunotherapy for metastatic melanoma Proc Am Soc Clin Oncol 2002 21 2790

[B63] Groenewegen G Bloem A De Gast GC Phase I/II study of sequential chemoimmunotherapy (SCIT) for metastatic melanoma: outpatient treatment with dacarbazine, granulocyte-macrophage colony-stimulating factor, low-dose interleukin-2, and interferon-alpha Cancer Immunol Immunother 2002 51 630 636 10.1007/s00262-002-0323-1 12439608 PMC11032800

[B64] de Gast GC Batchelor D Kersten MJ Vyth-Dreese FA Sein J van de Kasteele WF Nooijen WJ Nieweg OE de Waal MA Boogerd W Temozolomide followed by combined immunotherapy with GM-CSF, low-dose IL2 and IFN alpha in patients with metastatic melanoma Br J Cancer 2003 88 175 180 10.1038/sj.bjc.6600717 12610499 PMC2377058

[B65] Smith IJ Kurt RA Baher AG Denman S Justice L Doran T Gilbert M Alvord WG Urba WJ Immune effects of escalating doses of granulocyte-macrophage colony-stimulating factor added to a fixed, low-dose, inpatient interleukin-2 regimen: a randomized phase I trial in patients with metastatic melanoma and renal cell carcinoma J Immunother 2003 26 130 138 10.1097/00002371-200303000-00005 12616104

[B66] Lewis KD Gibbs P O’Day S Richards J Weber J Anderson C Zeng C Baron A Russ P Gonzalez R A phase II study of biochemotherapy for advanced melanoma incorporating temozolomide, decrescendo interleukin-2 and GM-CSF Cancer Invest 2005 23 303 308 10.1081/CNV-58832 16100942

[B67] Weber RW O’Day S Rose M Deck R Ames P Good J Meyer J Allen R Trautvetter S Timmerman M Cruickshank S Cook M Gonzalez R Spitler LE Low-dose outpatient chemobiotherapy with temozolomide, granulocyte-macrophage colony stimulating factor, interferon-alpha2b, and recombinant interleukin-2 for the treatment of metastatic melanoma J Clin Oncol 2005 23 8992 9000 10.1200/JCO.2005.02.5791 16260693

[B68] Gunturu KS Meehan KR Mackenzie TA Crocenzi TS McDermott D Usherwood EJ Margolin KA Crosby NA Atkins MB Turk MJ Ahonen C Fuse S Clark JI Fisher JL Noelle RJ Ernstoff MS Cytokine working group study of lymphodepleting chemotherapy, interleukin-2, and granulocyte-macrophage colony-stimulating factor in patients with metastatic melanoma: clinical outcomes and peripheral-blood cell recovery J Clin Oncol 2010 28 1196 1202 10.1200/JCO.2009.24.8153 20124177 PMC2834469

[B69] Lutzky J Lawson DH Enriquez-Nunez Y Gabrilovich D Phase II trial of high-dose interleukin-2 (IL-2) with priming and concomitant sargramostim (GM-CSF) in patients with advanced melanoma J Clin Oncol 2010 28 abstr 8560

[B70] Ali OA Emerich D Dranoff G Mooney DJ In situ regulation of DC subsets and T cells mediates tumor regression in mice Sci Transl Med 2009 1 8ra19 20368186 10.1126/scitranslmed.3000359 PMC2872791

[B71] Faries MB Hsueh EC Ye X Hoban M Morton DL Effect of granulocyte/macrophage colony-stimulating factor on vaccination with an allogeneic whole-cell melanoma vaccine Clin Cancer Res 2009 15 7029 7035 10.1158/1078-0432.CCR-09-1540 19903777 PMC2920049

[B72] Slingluff CL Jr Petroni GR Yamshchikov GV Barnd DL Eastham S Galavotti H Patterson JW Deacon DH Hibbitts S Teates D Neese PY Grosh WW Chianese-Bullock KA Woodson EM Wiernasz CJ Merrill P Gibson J Ross M Engelhard VH Clinical and immunologic results of a randomized phase II trial of vaccination using four melanoma peptides either administered in granulocyte-macrophage colony-stimulating factor in adjuvant or pulsed on dendritic cells J Clin Oncol 2003 21 4016 4026 10.1200/JCO.2003.10.005 14581425

[B73] Walter S Weinschenk T Stenzl A Zdrojowy R Pluzanska A Szczylik C Staehler M Brugger W Dietrich PY Mendrzyk R Hilf N Schoor O Fritsche J Mahr A Maurer D Vass V Trautwein C Lewandrowski P Flohr C Pohla H Stanczak JJ Bronte V Mandruzzato S Biedermann T Pawelec G Derhovanessian E Yamagishi H Miki T Hongo F Takaha N Multipeptide immune response to cancer vaccine IMA901 after single-dose cyclophosphamide associates with longer patient survival Nat Med 2012 18 1254 1261 10.1038/nm.2883 22842478

[B74] Chianese-Bullock KA Pressley J Garbee C Hibbitts S Murphy C Yamshchikov G Petroni GR Bissonette EA Neese PY Grosh WW Merrill P Fink R Woodson EM Wiernasz CJ Patterson JW Slingluff CL Jr MAGE-A1-, MAGE-A10-, and gp100-derived peptides are immunogenic when combined with granulocyte-macrophage colony-stimulating factor and montanide ISA-51 adjuvant and administered as part of a multipeptide vaccine for melanoma J Immunol 2005 174 3080 3086 10.4049/jimmunol.174.5.3080 15728523

[B75] Scheibenbogen C Schadendorf D Bechrakis NE Nagorsen D Hofmann U Servetopoulou F Letsch A Philipp A Foerster MH Schmittel A Thiel E Keilholz U Effects of granulocyte-macrophage colony-stimulating factor and foreign helper protein as immunologic adjuvants on the T-cell response to vaccination with tyrosinase peptides Int J Cancer 2003 104 188 194 10.1002/ijc.10961 12569574

[B76] Scheibenbogen C Schmittel A Keilholz U Allgauer T Hofmann U Max R Thiel E Schadendorf D Phase 2 trial of vaccination with tyrosinase peptides and granulocyte-macrophage colony-stimulating factor in patients with metastatic melanoma J Immunother 2000 23 275 281 10.1097/00002371-200003000-00012 10746554

[B77] Jager E Ringhoffer M Dienes HP Arand M Karbach J Jager D Ilsemann C Hagedorn M Oesch F Knuth A Granulocyte-macrophage-colony-stimulating factor enhances immune responses to melanoma-associated peptides in vivo Int J Cancer 1996 67 54 62 10.1002/(SICI)1097-0215(19960703)67:1<54::AID-IJC11>3.0.CO;2-C 8690525

[B78] Kirkwood JM Lee S Moschos SJ Albertini MR Michalak JC Sander C Whiteside T Butterfield LH Weiner L Immunogenicity and antitumor effects of vaccination with peptide vaccine+/−granulocyte-monocyte colony-stimulating factor and/or IFN-alpha2b in advanced metastatic melanoma: Eastern Cooperative Oncology Group Phase II Trial E1696 Clin Cancer Res 2009 15 1443 1451 10.1158/1078-0432.CCR-08-1231 19228745 PMC2759898

[B79] Soiffer R Hodi FS Haluska F Jung K Gillessen S Singer S Tanabe K Duda R Mentzer S Jaklitsch M Bueno R Clift S Hardy S Neuberg D Mulligan R Webb I Mihm M Dranoff G Vaccination with irradiated, autologous melanoma cells engineered to secrete granulocyte-macrophage colony-stimulating factor by adenoviral-mediated gene transfer augments antitumor immunity in patients with metastatic melanoma J Clin Oncol 2003 21 3343 3350 10.1200/JCO.2003.07.005 12947071

[B80] Soiffer R Lynch T Mihm M Jung K Rhuda C Schmollinger JC Hodi FS Liebster L Lam P Mentzer S Singer S Tanabe KK Cosimi AB Duda R Sober A Bhan A Daley J Neuberg D Parry G Rokovich J Richards L Drayer J Berns A Clift S Cohen LK Mulligan RC Dranoff G Vaccination with irradiated autologous melanoma cells engineered to secrete human granulocyte-macrophage colony-stimulating factor generates potent antitumor immunity in patients with metastatic melanoma Proc Natl Acad Sci USA 1998 95 13141 13146 10.1073/pnas.95.22.13141 9789055 PMC23738

[B81] Slingluff CL Jr Petroni GR Olson WC Smolkin ME Ross MI Haas NB Grosh WW Boisvert ME Kirkwood JM Chianese-Bullock KA Effect of granulocyte/macrophage colony-stimulating factor on circulating CD8+ and CD4+ T-cell responses to a multipeptide melanoma vaccine: outcome of a multicenter randomized trial Clin Cancer Res 2009 15 7036 7044 10.1158/1078-0432.CCR-09-1544 19903780 PMC2778314

[B82] Schaefer C Butterfield LH Lee S Kim GG Visus C Albers A Kirkwood JM Whiteside TL Function but not phenotype of melanoma peptide-specific CD8(+) T cells correlate with survival in a multiepitope peptide vaccine trial (ECOG 1696) Int J Cancer 2012 131 874 884 10.1002/ijc.26481 22021080 PMC3658448

[B83] Filipazzi P Valenti R Huber V Pilla L Canese P Iero M Castelli C Mariani L Parmiani G Rivoltini L Identification of a new subset of myeloid suppressor cells in peripheral blood of melanoma patients with modulation by a granulocyte-macrophage colony-stimulation factor-based antitumor vaccine J Clin Oncol 2007 25 2546 2553 10.1200/JCO.2006.08.5829 17577033

[B84] Parmiani G Castelli C Pilla L Santinami M Colombo MP Rivoltini L Opposite immune functions of GM-CSF administered as vaccine adjuvant in cancer patients Ann Oncol 2007 18 226 232 17116643 10.1093/annonc/mdl158

[B85] Harrington KJ Hingorani M Tanay MA Hickey J Bhide SA Clarke PM Renouf LC Thway K Sibtain A McNeish IA Newbold KL Goldsweig H Coffin R Nutting CM Phase I/II study of oncolytic HSV GM-CSF in combination with radiotherapy and cisplatin in untreated stage III/IV squamous cell cancer of the head and neck Clin Cancer Res 2010 16 4005 4015 10.1158/1078-0432.CCR-10-0196 20670951

[B86] Hu JCC Coffin RS Davis CJ A phase I study of OncoVEXGM-CSF, a second-generation oncolytic herpes simplex virus expressing granulocyte macrophage colony-stimulating factor Clin Cancer Res 2006 12 6737 6747 10.1158/1078-0432.CCR-06-0759 17121894

[B87] Kaufman HL Kim DW DeRaffele G Mitcham J Coffin RS Kim-Schulze S Local and distant immunity induced by intralesional vaccination with an oncolytic herpes virus encoding GM-CSF in patients with stage IIIc and IV melanoma Ann Surg Oncol 2010 17 718 730 10.1245/s10434-009-0809-6 19915919

[B88] Senzer NN Kaufman HL Amatruda T Nemunaitis M Reid T Daniels G Gonzalez R Glaspy J Whitman E Harrington K Goldsweig H Marshall T Love C Coffin R Nemunaitis JJ Phase II clinical trial of a granulocyte-macrophage colony-stimulating factor-encoding, second-generation oncolytic herpesvirus in patients with unresectable metastatic melanoma J Clin Oncol 2009 27 5763 5771 10.1200/JCO.2009.24.3675 19884534

[B89] Andtbacka RHI Collichio FA Amatruda T Senzer NN Chesney J Delman KA Spitler LE Puzanov I Doleman S Ye Y Vanderwalde AM Coffin R Kaufman H OPTiM: A randomized phase III trial of talimogene laherparepvec (T-VEC) versus subcutaneous (SC) granulocyte-macrophage colony-stimulating factor (GM-CSF) for the treatment (tx) of unresected stage IIIB/C and IV melanoma J Clin Oncol 2013 31 abstr LBA9008

[B90] Hodi FS Lee SJ McDermott DF Rao UNM Butterfield LH Tarhini AA Leming PD Puzanov I Kirkwood JM Multicenter, randomized phase II trial of GM-CSF (GM) plus ipilimumab (Ipi) versus Ipi alone in metastatic melanoma: E1608 J Clin Oncol 2013 31 abstr CRA9007

[B91] Heo J Breitbach CJ Moon A Kim CW Patt R Kim MK Lee YK Oh SY Woo HY Parato K Rintoul J Falls T Hickman T Rhee BG Bell JC Kirn DH Hwang TH Sequential therapy with JX-594, a targeted oncolytic poxvirus, followed by sorafenib in hepatocellular carcinoma: preclinical and clinical demonstration of combination efficacy Mol Ther 2011 19 1170 1179 10.1038/mt.2011.39 21427706 PMC3129795

[B92] Higano CS Corman JM Smith DC Centeno AS Steidle CP Gittleman M Simons JW Sacks N Aimi J Small EJ Phase 1/2 dose-escalation study of a GM-CSF-secreting, allogeneic, cellular immunotherapy for metastatic hormone-refractory prostate cancer Cancer 2008 113 975 984 10.1002/cncr.23669 18646045

[B93] Schoenfeld J Jinushi M Nakazaki Y Wiener D Park J Soiffer R Neuberg D Mihm M Hodi FS Dranoff G Active immunotherapy induces antibody responses that target tumor angiogenesis Cancer Res 2010 70 10150 10160 10.1158/0008-5472.CAN-10-1852 21159637 PMC3057563

[B94] Senzer N Barve M Kuhn J Melnyk A Beitsch P Lazar M Lifshitz S Magee M Oh J Mill SW Bedell C Higgs C Kumar P Yu Y Norvell F Phalon C Taquet N Rao DD Wang Z Jay CM Pappen BO Wallraven G Brunicardi FC Shanahan DM Maples PB Nemunaitis J Phase I trial of “bi-shRNAi(furin)/GMCSF DNA/autologous tumor cell” vaccine (FANG) in advanced cancer Mol Ther 2012 20 679 686 10.1038/mt.2011.269 22186789 PMC3293620

[B95] Adamina M Rosenthal R Weber WP Frey DM Viehl CT Bolli M Huegli RW Jacob AL Heberer M Oertli D Marti W Spagnoli GC Zajac P Intranodal immunization with a vaccinia virus encoding multiple antigenic epitopes and costimulatory molecules in metastatic melanoma Mol Ther 2010 18 651 659 10.1038/mt.2009.275 19935776 PMC2839441

[B96] Weide B Pascolo S Scheel B Derhovanessian E Pflugfelder A Eigentler TK Pawelec G Hoerr I Rammensee HG Garbe C Direct injection of protamine-protected mRNA: results of a phase 1/2 vaccination trial in metastatic melanoma patients J Immunother 2009 32 498 507 10.1097/CJI.0b013e3181a00068 19609242

[B97] Dillman RO Cornforth AN Depriest C McClay EF Amatruda TT de Leon C Ellis RE Mayorga C Carbonell D Cubellis JM Tumor stem cell antigens as consolidative active specific immunotherapy: a randomized phase II trial of dendritic cells versus tumor cells in patients with metastatic melanoma J Immunother 2012 35 641 649 10.1097/CJI.0b013e31826f79c8 22996370

[B98] Perales MA Yuan J Powel S Gallardo HF Rasalan TS Gonzalez C Manukian G Wang J Zhang Y Chapman PB Krown SE Livingston PO Ejadi S Panageas KS Engelhorn ME Terzulli SL Houghton AN Wolchok JD Phase I/II study of GM-CSF DNA as an adjuvant for a multipeptide cancer vaccine in patients with advanced melanoma Mol Ther 2008 16 2022 2029 10.1038/mt.2008.196 18797450 PMC3909666

